# Longitudinally Extensive Hemorrhagic Myelitis in a Young Adult With Newly Diagnosed Duodenal Adenocarcinoma

**DOI:** 10.7759/cureus.116743

**Published:** 2026-09-22

**Authors:** Izzathunnisa Rahmathullah, Janaka Peiris, Hemal Hordagoda, Srini Godevithanage, Nipuna Weerasinghe

**Affiliations:** 1 Neurology, The National Hospital Kandy, Kandy, LKA

**Keywords:** duodenal adenocarcinoma, hemorrhagic myelitis, longitudinally extensive myelitis, paraneoplastic myelopathy, spinal cord hemorrhage

## Abstract

Longitudinally extensive hemorrhagic myelitis is a rare clinical presentation with a broad differential diagnosis. We report a 20-year-old woman who presented with acute flaccid paraplegia, urinary retention, and a T2 sensory level. Magnetic resonance imaging demonstrated longitudinally extensive intramedullary spinal cord involvement with hemorrhagic features extending from C4 to the conus medullaris. Cerebrospinal fluid showed inflammatory changes and xanthochromia. Aquaporin-4 and myelin oligodendrocyte glycoprotein antibodies, oligoclonal bands, and tumor markers were negative. Further evaluation revealed a circumferential lesion in the second part of the duodenum, with histology confirming adenocarcinoma. No metastatic disease was identified. She received corticosteroids, plasma exchange, and intravenous immunoglobulin, followed by extensive surgical resection of the tumor. Neurological recovery remained poor. In the absence of another clear cause, the clinical and radiological findings raised the possibility of a paraneoplastic inflammatory myelopathy.

## Introduction

Longitudinally extensive transverse myelitis (LETM) refers to spinal cord involvement extending over three or more vertebral segments. It is commonly associated with inflammatory demyelinating disorders, particularly neuromyelitis optica spectrum disorder (NMOSD) and myelin oligodendrocyte glycoprotein antibody-associated disease (MOGAD) [1,2]. However, LETM can also occur with infections, systemic autoimmune disease, vascular disorders, neoplastic infiltration, and paraneoplastic neurological syndromes.

Hemorrhagic involvement of the spinal cord is uncommon in inflammatory myelitis. When hemorrhage is present, vascular and other non-demyelinating causes need to be considered. Hemorrhagic LETM has been reported as a rare and severe presentation, with several possible underlying causes [3,4].

Paraneoplastic myelopathy is an uncommon neurological manifestation of malignancy. It may present with longitudinally extensive spinal cord abnormalities, inflammatory cerebrospinal fluid findings, and progressive neurological dysfunction. In some patients, the neurological syndrome may precede the diagnosis of the underlying cancer [5-7].

We report a young woman who presented with acute hemorrhagic LETM and was subsequently diagnosed with duodenal adenocarcinoma. The combination of the clinical presentation, spinal imaging, inflammatory cerebrospinal fluid findings, and absence of another clear cause raised the possibility of a paraneoplastic inflammatory myelopathy.

## Case presentation

A 20-year-old woman presented with sudden-onset bilateral lower limb numbness and weakness. Her symptoms progressed rapidly over 48 hours, resulting in flaccid paraplegia, urinary retention, and absent lower limb reflexes. Examination revealed a T2 sensory level with sphincter involvement. At initial presentation, the upper limbs were neurologically intact, with normal proximal and distal power and easily elicited, symmetrical biceps and triceps reflexes. There was no upper-limb sensory deficit, and respiratory and bulbar functions were preserved. However, subsequently she developed significant bilateral hand weakness with an exaggerated supinator jerk, suggesting later cervical cord involvement. Despite this, the lower limbs remained persistently flaccid and areflexic, without development of spasticity. Respiratory, bulbar functions, and other cranial nerves remained preserved throughout the illness.

She had a history of a rectal tubulovillous adenoma diagnosed at the age of 17, for which she underwent annual colonoscopic surveillance with intermittent polypectomies. She was the only child in her family, and there was no family history suggestive of an inherited gastrointestinal polyposis syndrome. Serial colonoscopic findings were not suggestive of familial adenomatous polyposis, and genetic testing had not been performed.

Magnetic resonance imaging of the spine demonstrated a longitudinally extensive intramedullary T2 hyperintense signal abnormality extending from C4 to the conus medullaris and involving almost the entire cross-section of the spinal cord. Lobulated and elongated areas of heterogeneous T1- and T2-weighted signal intensity were seen within the affected cord, involving the cervical and upper dorsal regions and the lower dorsal cord and conus medullaris separately. The spinal cord was expanded in these regions. These abnormal areas contained foci of profoundly low T1- and T2-weighted signal intensity with predominantly peripheral, moderately patchy enhancement. The inferior abnormality extended beyond the conus medullaris into the cauda equina nerve roots up to the L3 vertebral level, predominantly on the right side. A fluid-fluid level was also seen within the thecal sac at the S1 level, reported as indicative of subarachnoid bleeding. There was no focal mass or extrinsic compression of the spinal cord (Figures 1, 2).

**Figure 1:**
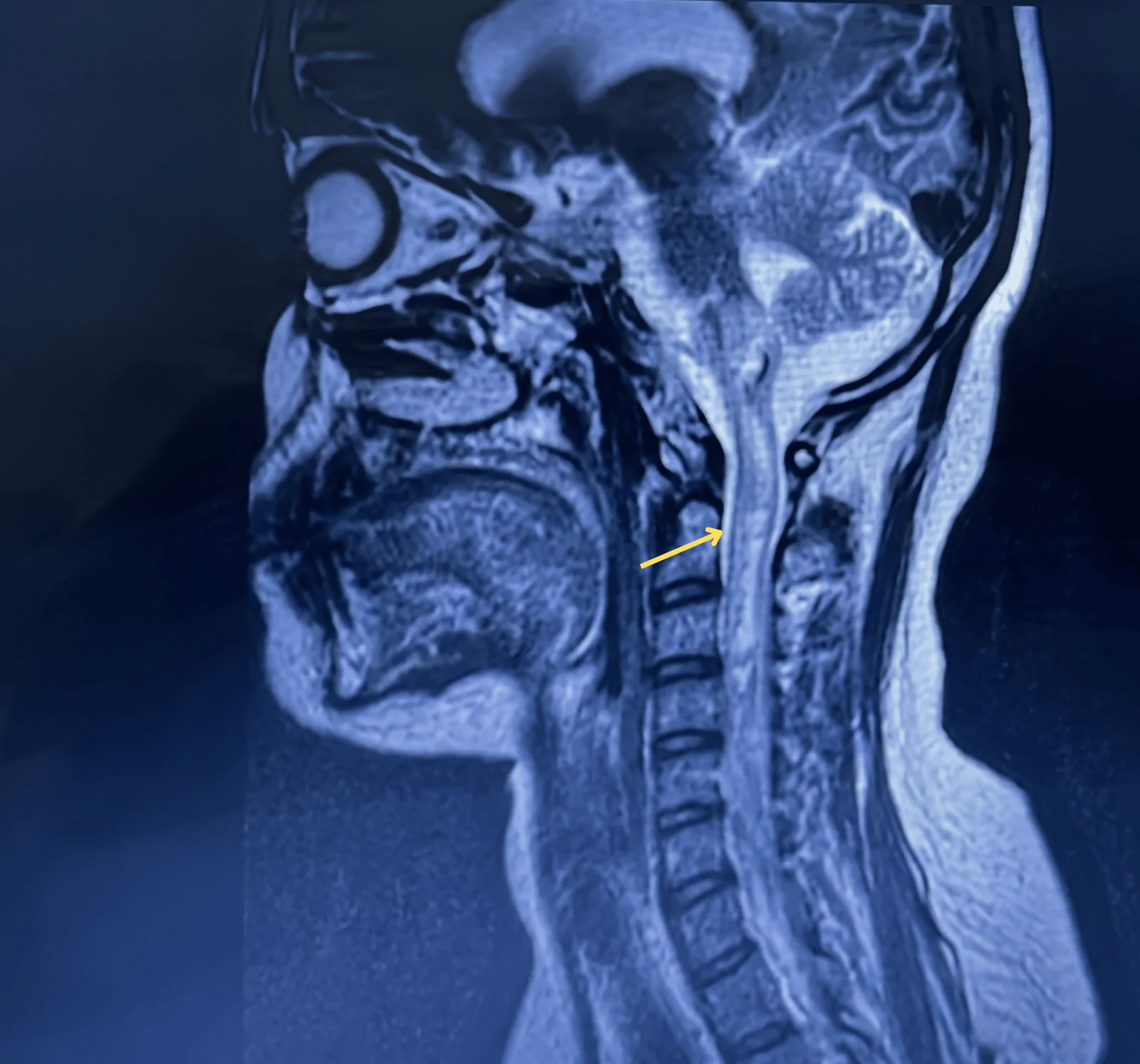
Sagittal T2-weighted MRI demonstrating longitudinally extensive intramedullary spinal cord hyperintensity

**Figure 2:**
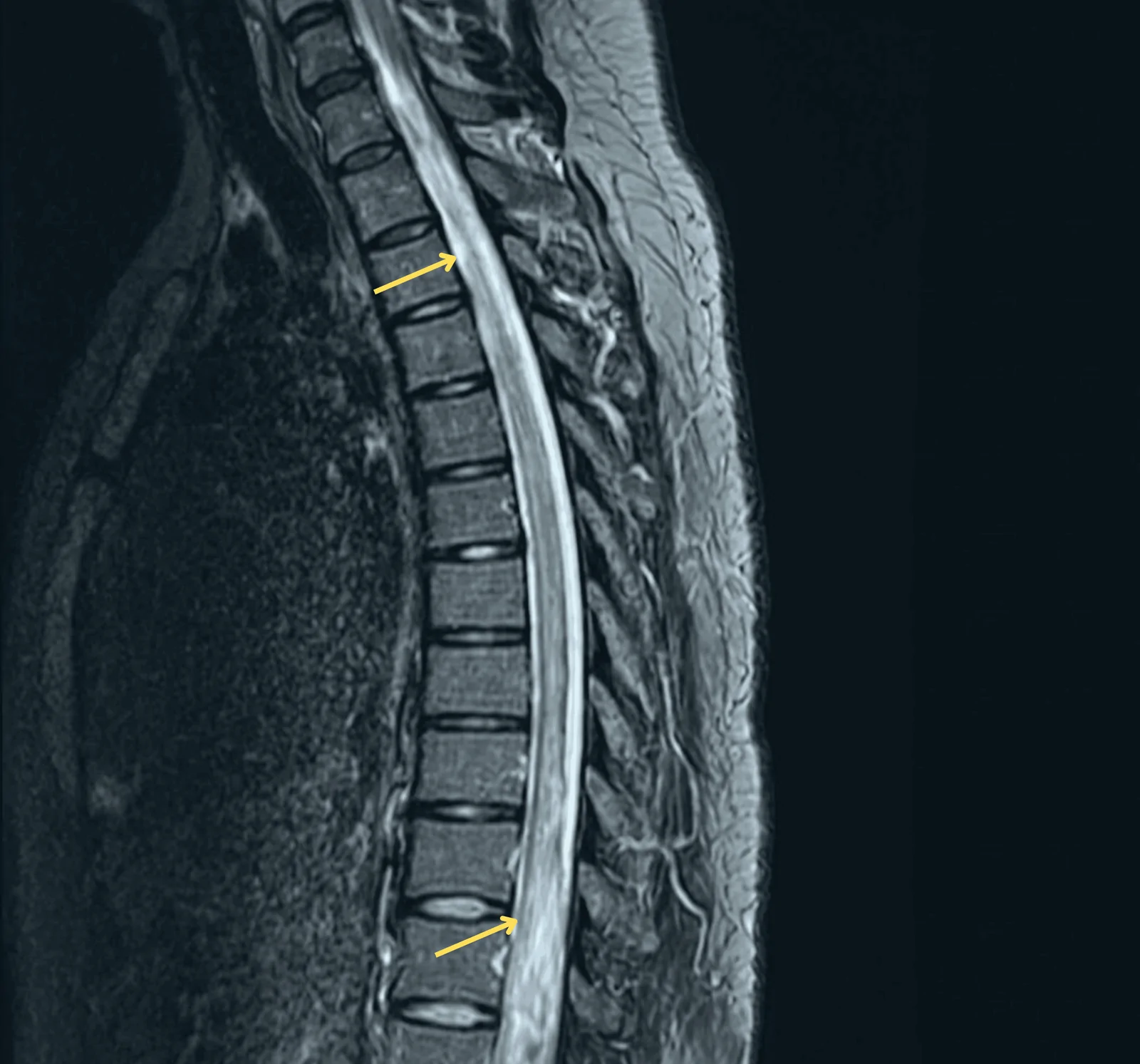
Sagittal T2-weighted MRI demonstrating longitudinally extensive intramedullary hyperintensity of the thoracic spinal cord

Cerebrospinal fluid (CSF) examination showed a blood-stained, xanthochromic appearance, with a white blood cell count of 17 cells/µL (35% polymorphs and 65% lymphocytes; reference value: 0-3 cells/µL), red blood cell count of 8,167 cells/µL (normally absent), protein of 399.7 mg/dL (reference value: 20-45 mg/dL), and glucose of 1.9 mmol/L (reference value: 2.78-5.55 mmol/L), with a simultaneous serum glucose of 6.2 mmol/L (Figure 3).

**Figure 3:**
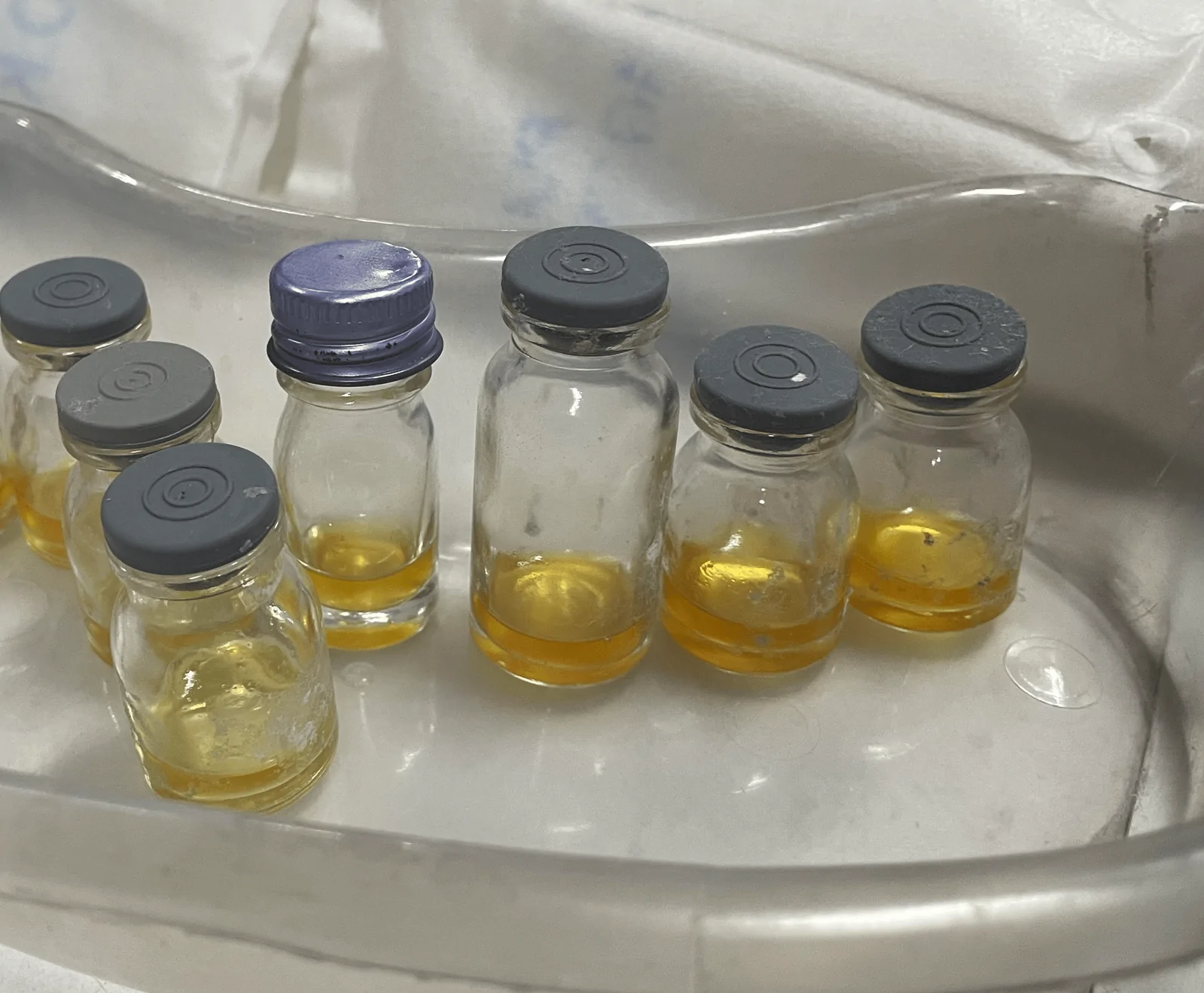
Xanthochromic cerebrospinal fluid with blood-stained supernatant

The opening pressure was normal. CSF culture showed no growth, CSF Venereal Disease Research Laboratory (VDRL) was non-reactive, acid-fast bacilli testing was negative, and CSF polymerase chain reaction (PCR), including tuberculosis PCR, was negative. CSF cytology was negative; flow cytometry was not performed. The systemic infectious work-up, including blood cultures, sputum examination, and testing for tuberculosis, syphilis, hepatitis B, cytomegalovirus, Epstein-Barr virus, retroviral infection, poliovirus, enterovirus, and other relevant viral infections, was negative. Oligoclonal bands were negative. Serum aquaporin-4 immunoglobulin G and anti-myelin oligodendrocyte glycoprotein antibodies were negative. Tumor markers were within normal limits. However, a paraneoplastic antibody panel was not performed.

During the subsequent clinical course, the patient developed dark stools with worsening anemia. Contrast-enhanced CT of the abdomen demonstrated an irregularly enhancing endopancreatic lesion involving the proximal second part of the duodenum, causing approximately 75% luminal obstruction, without significant lymphadenopathy. Upper gastrointestinal endoscopy demonstrated a circumferential lesion in the second part of the duodenum. Histopathological assessment reported a grade 2 (G2) adenocarcinoma [8] with intramucosal involvement. Repeat lower gastrointestinal endoscopy did not show significant recurrent adenomatous lesions. Germline genetic testing was not performed. Contrast-enhanced CT of the chest, abdomen, and pelvis showed no evidence of metastatic disease.

The main differential diagnoses included NMOSD, MOGAD, spinal vascular malformation, intramedullary neoplasm or metastasis, and infective myelitis. NMOSD and MOGAD were considered less likely because of the negative antibody testing and the hemorrhagic imaging features. A focal intramedullary tumor or metastatic lesion was not identified, and there was no evidence of systemic metastatic disease. Although a vascular etiology was considered given the acute onset and hemorrhagic spinal cord abnormality, no focal vascular lesion was identified on the available imaging. However, a spinal vascular malformation could not be definitively excluded because dedicated spinal vascular imaging was not performed The inflammatory cerebrospinal fluid findings also suggested an inflammatory process. There were no clinical features or investigations supporting an active systemic infection.

The patient received intravenous methylprednisolone at a dose of 1 g daily for five days, followed by plasma exchange, with five sessions performed on alternate days. As there was no significant neurological improvement, intravenous immunoglobulin was subsequently administered at a dose of 0.4 g/kg. The duodenal adenocarcinoma was diagnosed approximately one month after the initial neurological presentation. She underwent Whipple's pancreaticoduodenectomy with total colectomy three months after the initial presentation. Following surgery, she was followed for a further six months. Despite tumor-directed treatment, there was no significant improvement in motor power. The lower limbs remained flaccid and areflexic, while progressive weakness of the upper limbs developed. Bladder dysfunction persisted, and she remained bed-bound and functionally dependent until the time of reporting. Written informed consent for publication of the patient's clinical details and accompanying images was obtained from the patient.

## Discussion

LETM is a radiological pattern rather than a specific diagnosis. In a young patient, NMOSD and MOGAD are important considerations, particularly when the spinal cord lesion extends over several vertebral segments [1,2]. In our patient, however, the hemorrhagic component was unusual for these disorders. Hemorrhagic LETM has been described as a rare and severe presentation and may occur in association with inflammatory, autoimmune, or other mechanisms [3,4].

The hemorrhagic component also led us to consider a vascular cause. Spinal vascular malformations, including dural arteriovenous fistula, can produce extensive spinal cord abnormalities and may be difficult to distinguish from inflammatory myelitis. In this case, no focal vascular abnormality was identified on the available imaging. The inflammatory CSF findings and the clinical context also made a purely structural vascular lesion less likely.

Direct involvement of the spinal cord by malignancy was another important consideration after the duodenal adenocarcinoma was diagnosed. However, there was no discrete intramedullary mass or spinal cord compression, and staging CT did not show metastatic disease. These findings argued against direct malignant infiltration as the cause of the spinal cord lesion.

Paraneoplastic myelopathy is rare but should be considered when myelopathy has an unusual clinical or radiological pattern and an underlying malignancy is identified. In a series of 31 patients with isolated paraneoplastic myelopathy, longitudinally extensive spinal cord lesions were found in 14 patients, while the neurological syndrome preceded the cancer diagnosis in 18 patients. Neurological recovery was limited in many patients despite treatment [5]. Another study of patients with paraneoplastic myelopathy also reported inflammatory CSF abnormalities and longitudinally extensive spinal cord lesions, with generally poor treatment responses [7].

The updated diagnostic criteria for paraneoplastic neurological syndromes emphasize that the diagnosis requires a combination of the neurological phenotype, cancer association, antibody findings, and exclusion of alternative causes [6]. In our patient, a paraneoplastic antibody panel was not performed. Therefore, a definite paraneoplastic neurological syndrome cannot be established. The relationship between the myelitis and the newly diagnosed duodenal adenocarcinoma should be considered a clinical possibility rather than a proven causal association.

The hemorrhagic component adds another unusual feature to this case. A previous case report and literature review described acute hemorrhagic LETM as an uncommon and severe form of spinal cord inflammation [4]. The presence of blood products within the cord may reflect vascular injury occurring in the setting of an inflammatory process, although the exact mechanism remains uncertain in our patient.

Duodenal adenocarcinoma is itself an uncommon malignancy. Small bowel adenocarcinomas are rare, with the duodenum being the most frequent primary site [8]. They are usually diagnosed in older adults, making the diagnosis in a 20-year-old particularly unusual. Predisposing conditions include several inherited gastrointestinal syndromes, although most cases are sporadic [8,9]. In our patient, the previous tubulovillous adenoma and young age raised the question of an underlying predisposition, but the clinical and endoscopic findings were not suggestive of familial adenomatous polyposis and genetic testing was not performed.

The coexistence of duodenal adenocarcinoma and hemorrhagic LETM does not, by itself, prove a paraneoplastic relationship. Nevertheless, the absence of another clear cause, the inflammatory nature of the CSF, the extensive hemorrhagic spinal cord lesion, and the temporal identification of an occult malignancy provide a reasonable basis for considering a paraneoplastic inflammatory myelopathy.

The poor neurological recovery despite corticosteroids, plasma exchange, intravenous immunoglobulin, and tumor resection is also noteworthy. Severe paraneoplastic myelopathy may result in irreversible spinal cord injury, particularly when treatment is started after substantial neurological damage has occurred [5,7]. However, the lack of neurological improvement after tumor resection means that a therapeutic response cannot be used to confirm the proposed paraneoplastic mechanism in this case.

This case has several limitations. Paraneoplastic antibody testing was not performed, and therefore there is no serological evidence supporting a specific paraneoplastic syndrome. In addition, the absence of neurological improvement following tumor resection does not provide evidence for or against causality. Nevertheless, the case demonstrates the diagnostic challenge posed by hemorrhagic LETM and the importance of considering an underlying malignancy when the clinical and radiological findings do not fit a typical inflammatory demyelinating disorder.

## Conclusions

Hemorrhagic LETM requires consideration of inflammatory, vascular, infectious, neoplastic, and paraneoplastic causes. In this patient, the extensive hemorrhagic spinal cord lesion, inflammatory cerebrospinal fluid findings, negative aquaporin-4 and myelin oligodendrocyte glycoprotein antibodies, absence of metastatic disease, and newly diagnosed duodenal adenocarcinoma raised the possibility of a paraneoplastic inflammatory myelopathy.

Although a causal relationship could not be established, this case highlights that an underlying malignancy may be considered in selected patients with atypical or unexplained myelitis following comprehensive evaluation. The temporal association between hemorrhagic LETM and duodenal adenocarcinoma in this patient may represent a paraneoplastic process, although other inflammatory, vascular, infectious, and neoplastic causes should also be considered.
